# Machine Learning and First-Principle Predictions of Materials with Low Lattice Thermal Conductivity

**DOI:** 10.3390/ma17215372

**Published:** 2024-11-02

**Authors:** Chia-Min Lin, Abishek Khatri, Da Yan, Cheng-Chien Chen

**Affiliations:** 1 Department of Physics, University of Alabama at Birmingham, Birmingham, AL 35294, USA; cleanfreexyz@gmail.com (C.-M.L.); khatria@uab.edu (A.K.); 2 Department of Computer Sciences, Indiana University Bloomington, Bloomington, IN 47405, USA; yanda@iu.edu

**Keywords:** lattice thermal conductivity, thermoelectric material, machine learning, density functional theory

## Abstract

We performed machine learning (ML) simulations and density functional theory (DFT) calculations to search for materials with low lattice thermal conductivity, $\kappa_{L}$. Several cadmium (Cd) compounds containing elements from the alkali metal and carbon groups including A_2_CdX (A = Li, Na, and K; X = Pb, Sn, and Ge) are predicted by our ML models to exhibit very low $\kappa_{L}$ values (<1.0 W/mK), rendering these materials suitable for potential thermal management and insulation applications. Further DFT calculations of electronic and transport properties indicate that the figure of merit, ZT, for the thermoelectric performance can exceed 1.0 in compounds such as K_2_CdPb, K_2_CdSn, and K_2_CdGe, which are therefore also promising thermoelectric materials.

## 1. Introduction

Materials with low lattice thermal conductivity ($\kappa_{L}$) have important applications in thermal management and energy conversion by serving as thermal insulation and barrier coatings, or as thermoelectrics. In particular, thermoelectric (TE) materials can directly convert between thermal and electrical energy [1,2,3,4,5,6,7,8], offering potential solutions for sustainable clean energy. To date, however, large-scale TE applications remain limited due to the relatively low energy conversion efficiency of known materials. Improving their efficiency and finding suitable TE materials that function at different temperatures remain important tasks in materials science research.

The TE performance can be quantified by the figure of merit value $ZT=S^{2}\sigma T/\kappa$, where *S* is the Seebeck coefficient (the induced voltage in response to a temperature gradient), σ is the electrical conductivity, *T* is the temperature, and κ is the thermal conductivity. One approach to enhance ZT is by increasing the power factor ($S^{2}\sigma$) through the band engineering of carrier concentration and mobility, among other factors [9,10,11,12,13,14,15]. Another approach is to find materials with low thermal conductivity [16,17,18].

There are two major contributions to thermal conductivity: $\kappa =\kappa_{e}+\kappa_{L}$. In general, the electronic contribution $\kappa_{e}$ closely follows the Wiedemann–Franz law [19], $\kappa_{e}=L\sigma T$, where *L* is the Lorenz number ($2.44\times 10^{-8}$ WΩ/K^2^ for free electrons). $\kappa_{e}$ also varies with the charge carrier concentration *n*. On the other hand, $\kappa_{L}$ has a distinct *T* dependence. If the lattice contribution $\kappa_{L}$ of a material is much lower than the electronic contribution $\kappa_{e}$ under certain *n* and *T* conditions, an optimal $ZT∼S^{2}/L>1$ can be achieved due to the Wiedemann–Franz law. Therefore, designing or searching for materials with low $\kappa_{L}$ continues to be an active research area employing approaches such as in phonon engineering, nanostructuring, and/or applying external strain or pressure [20,21,22,23,24,25,26].

Computational materials modeling has played an important role in providing predictions and critical insights into the thermal conducting behavior of materials [27,28,29,30,31,32]. Traditionally, density functional theory (DFT) is the standard computational workforce for accurate calculations of $\kappa_{L}$ from first principles. However, its relatively high computational cost limits large-scale investigations of $\kappa_{L}$ in new materials. More recently, data-driven machine learning (ML) approaches have become popular and powerful tools for materials modeling and discovery [33,34,35,36,37,38,39,40,41,42,43,44,45]. This popularity and improvement in ML research largely result from advancements in computer architectures and ML algorithms, as well as from the increasing availability of open materials databases. ML algorithms can learn from training data by identifying connections through linear or non-linear relationships between target properties and input features. Once trained, ML models can achieve highly efficient and often accurate large-scale predictions.

In this study, we utilized combined machine learning (ML) predictions and density functional theory (DFT) calculations to discover materials with low lattice thermal conductivity. Specifically, we developed ensemble tree ML models with input features based on chemical formulas and atomic configurations to quickly estimate the $\kappa_{L}$ of a given material. For promising low-κ materials identified by our ML models, we further validated the results by performing DFT calculations to evaluate $\kappa_{L}$ directly from first principles. In particular, we found that the chemical compositions A_2_CdX (A = Li, Na, and K; X = Pb, Sn, and Ge) of orthorhombic crystal symmetry exhibit *ultra-low lattice thermal conductivity* ($\kappa_{L}∼$ 0.1–1.0 W/mK). Our DFT calculations of the transport and thermoelectric properties further indicate that some of these materials like K_2_CdPb can exhibit a ZT≥1.0 near room temperature, and are therefore promising for *low-temperature thermoelectric applications* [46].

The rest of this paper is organized as follows: Section 2 presents the computational details of machine learning (ML) models and first-principle density functional theory (DFT) calculations. Section 3 presents the ML and DFT predictions of low-$\kappa_{L}$ materials and their thermoelectric properties. Finally, Section 4 concludes the paper by summarizing our main findings.

## 2. Computational Methods

### 2.1. Machine Learning Simulation

*Data Acquisition and Feature Creation*—Our machine learning (ML) models aim to predict the target property of lattice thermal conductivity $\kappa_{L}$ for a given compound. The ML training dataset was sourced from the TE Design Lab, which is a virtual platform hosting a database of calculated thermoelectric properties [47]. From this database, we selected a total of 1900 compounds with $\kappa_{L}$ in the range of 0–1100 W/mK. For all compounds in the selected dataset, we then used theMatminer package (version 0.7.8) [48] to generate 61 input features based on their chemical formulas and atomic configurations [49]. These features can be broadly categorized as structural features and elemental features. Specifically, seven structural features include the space group, volume per atom, packing fraction, unit-cell density, bond length, bond angle, and cohesive energy. Moreover, 18 elemental features include the atomic mass, atomic radius, atomic number, periodic table group, row number, block number, Mendeleev number, molar volume, boiling point, melting temperature, Pauling electronegativity, first ionization energy, covalent radius, and volume per atom from ground state, as well as the average number of *s*, *p*, *d*, and *f* valence electrons. Since our dataset contains compounds ranging from unary to quinary materials, each elemental feature can be expanded by calculating the minimum, maximum, and weighted average of the constituent chemicals, resulting in a total of 54 (=18 × 3) elemental features. Overall, 61 (=7 + 54) features were used in the training of the ML models.

We note that several features, such as average atomic mass and volume (which is related to atomic radius), are relevant parameters for estimating $\kappa_{L}$ in known empirical formulas [50,51]. Therefore, $\kappa_{L}$ is also expected to be proportional to the mean sound velocity $v_{m}$ (or the Debye temperature $\Theta_{D}$) cubed. It has been shown that ML models can accurately predict $v_{m}$ and $\Theta_{D}$ using features simply derived from chemical compositions and crystal symmetry [52]. Therefore, it was anticipated that the ML models trained here with the 61 features under study could perform well in predicting $\kappa_{L}$ [49].

*Model Training and Validation*—Our supervised ML tasks utilized Random Forest as the underlying algorithm [53,54]. Random Forest is an ensemble method consisting of multiple decision trees. Each tree is trained on a randomly selected subset of features and samples. The Random Forest algorithm then averages the results of all trees to make the final prediction, which generally reduces the overfitting problem associated with a single decision tree. Random Forest ML models are relatively easy to train and often produce highly accurate results. To further reduce overfitting, we also pre-pruned the trees by limiting their depth. Specifically, we used 90% of our input data as the training–validation set and applied the GridSearchCV technique from the scikit-learn library [55] to determine the optimal tree depth via 10-fold cross-validation. The remaining 10% of the input data served as the unbiased test set to evaluate the final model performance. After training and evaluation, we then used the ML model to predict the lattice thermal conductivity $\kappa_{L}$.

### 2.2. First-Principle Calculation

For promising low-$\kappa_{L}$ materials identified by ML models, we further performed first-principle density functional theory (DFT) calculations to validate their thermoelectric properties. Our calculations are based on the Vienna Ab initio Simulation Package (VASP, version 5.4.4) [56,57], which is a highly efficient and accurate plane-wave pseudopotential DFT code. We adopted the projector augmented wave (PAW) potentials [58,59] and utilized the Perdew–Burke–Ernzerhof generalized gradient approximation (GGA-PBE) functional [60]. The plane-wave cutoff energy was set to 500 eV, and a fine Γ-centered Monkhorst–Pack grid of 19×19×19 points was used for Brillouin zone integration [61]. For a given crystal structure, we first fully relaxed the lattice parameters and atomic positions. The convergence criteria for the electronic and ionic relaxation loops were set to $10^{-8}$ eV per unit cell and $10^{-4}$ eV/Å, respectively.

After structure relaxation, we computed the thermoelectric properties (*S*, σ, and $\kappa_{e}$) using theBoltzTraP2 package (version 20.7.1) [62], which is based on Boltzmann transport theory with a constant relaxation time approximation. The lattice thermal conductivity ($\kappa_{L}$) was obtained through first-principle phonon calculations using the Phonopy (version 2.11.0) and Phono3py (version 2.4.0) [63,64] packages, which are based on finite-displacement supercell approaches. Phonopy computes the phonon spectra at the harmonic or quasi-harmonic level. Phono3py evaluates phonon–phonon interactions and $\kappa_{L}$ from the Peierls–Boltzmann equation [65]. In the supercell calculations, the atomic displacement was set to 0.02 Å, and the real-space interaction cutoff distance was set to 4.0 Å. For the second-order (harmonic) and third-order (anharmonic) phonon calculations, 3×3×3 supercells with a 5×5×5 k-mesh and 2×2×2 supercells with a 9×9×9 k-mesh were employed, respectively. A phonon q-point sampling mesh of 21×21×21 points was used. The theoretical crystal structure in this study was visualized using VESTA software (version 3.4.8) [66].

## 3. Results and Discussion

**Figure 1:**
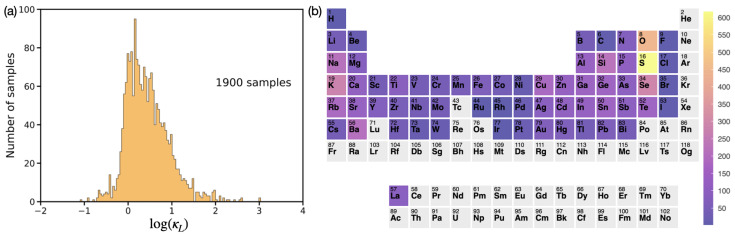
(**a**) Histogram for the 1900 training samples of lattice thermal conductivity ($\kappa_{L}$) selected from the TE Design Lab [47]. The distribution spans nearly five orders of magnitude and is plotted on a base-10 logarithmic scale. (**b**) False-color intensity plot showing the frequency of each element in the 1900-sample training dataset. Elements not present in the list are shown in gray. The figure was created using the open-source software Periodic Trend Plotter (accessed on 1 November 2024) [67].

Figure 1a shows the distribution of $\kappa_{L}$ for the 1900 compounds in our training dataset from the TE Design Lab [47]. Since the range of the distribution spans nearly five orders of magnitude, it is plotted on a base-10 logarithmic scale. Eventually, ML models were trained to predict $\log(\kappa_{L})$. For the accuracy and generalizability of our ML models, we ensured that our dataset was diverse in chemical composition (from unary to quinary compounds) and crystal structure (with 140 different space groups). In particular, among the 1900 samples, 7 were unary, 418 were binary, 1143 were ternary, 328 were quaternary, and 4 were quinary. These compounds contained 61 different atomic elements. The frequency of each element appearing in the compound list is represented by the false-color intensity plot in Figure 1b; gray means that the element is not present.

**Figure 2:**
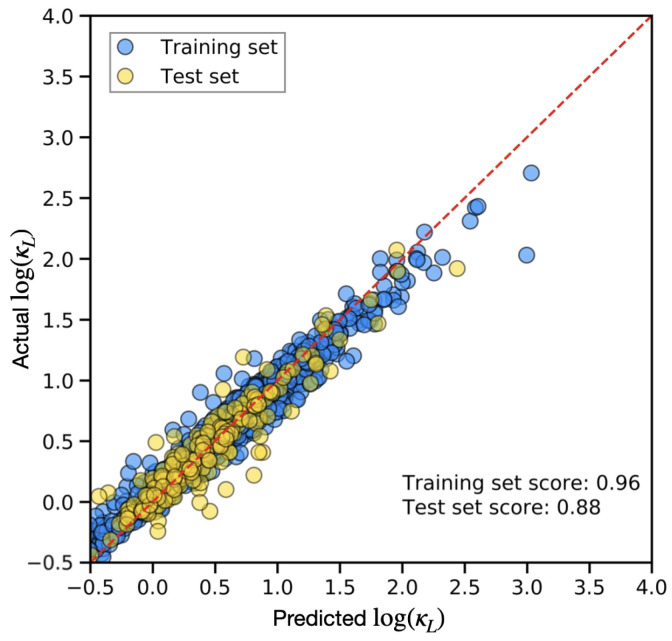
EvaluationMDPI: The 0.5 below 0.0 is missing a minus sign, please modify the image. of the Random Forest model in predicting the logarithmic value of lattice thermal conductivity, $\log(\kappa_{L})$. The training and test sets consist of 90% and 10% of our total dataset (1900 samples), respectively. When the machine learning prediction perfectly matches the actual value, the data point will fall on the red dashed line. The model achieved relatively high $R^{2}$ scores of 0.96 and 0.88 for the training and test sets, respectively.

As discussed in Section 2, our ML models are based on Random Forest trained with 61 features [49] generated by the Matminer package [48]. The coefficient of determination $R^{2}$ was used to evaluate the model performance:

(1) $$\begin{array}{c} R^{2}=1-\frac{\sum_{i}{\left(y_{i}-\hat{y_{i}}\right)}^{2}}{\sum_{i}{\left(y_{i}-\bar{y}\right)}^{2}}, \end{array}$$

where $y_{i}$, $\hat{y_{i}}$, $\bar{y}$ are the actual value (for the *i*-th entry), the predicted value, and the mean of the actual values, respectively. $R^{2}$ ranges from 0 to 1, with $R^{2}=1$ indicating a perfect prediction. Figure 2 shows the resulting ML model performance on predicting $\log(\kappa_{L})$. The blue and yellow circles represent data from the training–validation set (90%) and the test set (10%), respectively. A red dashed line is also plotted as a guide to the ideal line where the predicted values match the actual values. Our model achieved an $R^{2}=0.96$ for the training–validation set and $R^{2}=0.88$ for the test set, indicating that our ML model provides a fairly accurate prediction of $\log(\kappa_{L})$.

Random Forest models also provide information on feature importance in ML predictions. Among the features under study, the atomic bond length was found to be the most significant factor affecting $\kappa_{L}$. In an ideal gas model, lattice thermal conductivity is approximated as

(2) $$\kappa_{L}=\frac{1}{3}v_{s}^{2}c_{v}\tau_{s},$$

where $v_{s}$ is the phonon velocity, $c_{v}$ is the specific heat, and $\tau_{s}$ is the phonon relaxation time. Among these three parameters, the phonon relaxation time is related to the bond-strength anharmonicity [68,69,70], which is correlated with bond length. In particular, a longer bond length is prone to causing anharmonic vibrations, as the interatomic force constant decreases with increasing bond length. Anharmonicity then facilitates collisions between different phonon modes. As anharmonicity increases, the phonon relaxation time decreases, which in turn leads to a reduction in lattice thermal conductivity.

We note that the strength of anharmonicity can also be evaluated with the Grüneisen parameter:

(3) $$\gamma =\frac{V}{\omega }\frac{\partial \omega }{\partial V},$$

where *V* is the crystal volume, and ω is the phonon frequency. Within the harmonic approximation, the thermal expansion is zero on average. In the presence of anharmonicity, the phonon frequency can vary as the volume changes with temperature. Therefore, a larger Grüneisen parameter indicates stronger anharmonicity and a lower lattice thermal conductivity. In fact, based on the Debye–Callaway model [50,51], the lattice thermal conductivity can be approximately evaluated as

(4) $$\kappa_{L}\approx \frac{Mv_{m}^{3}}{TV^{2/3}\gamma^{2}}\frac{1}{N^{1/3}},$$

where *M*, $v_{m}$, *T*, *V*, γ, and *N* represent the average mass, the mean speed of sound, the temperature, the average volume per atom, the Grüneisen parameter, and the number of atoms per primitive unit cell, respectively. The above formula shows that $\kappa_{L}$ is inversely proportional to $\gamma^{2}$ and $V^{2/3}$. Indeed, in addition to bond length, the volume per atom is evaluated by our ML models as the second most important feature affecting $\kappa_{L}$. Overall, the feature importance values align well with the above approximated models for $\kappa_{L}$, demonstrating that our ML models are reasonable and adequate.

**Figure 3:**
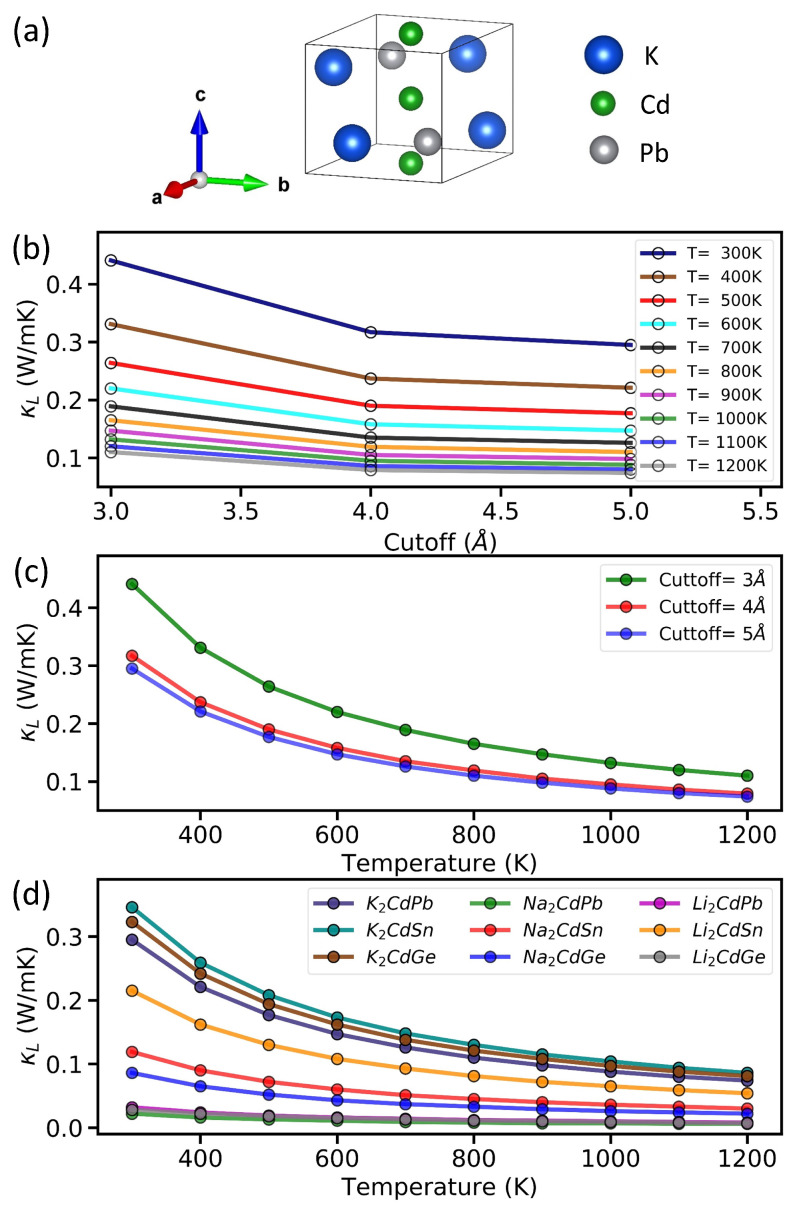
(**a**) Primitive-cell crystal structure of K_2_CdPb, with orthorhombic symmetry and space group Ama2 (No. 40). (**b**) Lattice thermal conductivity $\kappa_{L}$ of K_2_CdPb as a function of the neighbor interaction cutoff distance in the temperature range of 300–1200 K. (**c**) $\kappa_{L}$ of K_2_CdPb as a function of temperature for different cutoff distances. (**d**) $\kappa_{L}$ computed with a cutoff distance of 4 Å for nine different Cd compounds.

We then applied the ML models to predict materials with low $\kappa_{L}$. Recently, Zintl-phase compounds have attracted significant attention due to their strong anharmonic properties, which could lead to low lattice thermal conductivity [71,72,73,74,75,76,77,78]. The Zintl phase refers to compounds formed by alkali metals (group I) or alkaline earth metals (group II) combined with *p*-block metals or metalloids (from groups III–VI). Other recent studies have also shown that Zintl-phase compounds can achieve ultra-low $\kappa_{L}$ by introducing a heavy element, cadmium (Cd) [20,79]. For these reasons, we focused on applying our ML models to Cd-based Zintl-phase materials. Specifically, we considered A_2_CdX (A = Li, Na, and K; X = Pb, Sn, and Ge) with orthorhombic symmetry and space group Ama2 (No. 40) [80]; Figure 3a shows the corresponding crystal structure for K_2_CdPb. As seen in Table 1, the $\kappa_{L}$ values predicted by our ML models for the nine compositions A_2_CdX (A = Li, Na, and K; X = Pb, Sn, and Ge) range from 0.69 to 0.95 W/mK, indicating that all these compounds are potential low-$\kappa_{L}$ materials.

To validate the ML predictions, we further performed first-principle calculations to directly compute $\kappa_{L}$ and other thermoelectric properties for the nine compounds under study. We first focused on K_2_CdPb [80], for which its primitive cell structure is shown in Figure 3a. To ensure an accurate calculation of $\kappa_{L}$, we conducted a convergence test for the neighbor interaction cutoff distance. Figure 3b,c show the convergence tests for K_2_CdPb as functions of temperature and cutoff distance. Notably, the $\kappa_{L}$ computed with a cutoff of 4 Å is very close to the result obtained with a 5 Å cutoff. Therefore, for both accuracy and efficiency considerations, we adopted a cutoff distance of 4 Å for the other compounds. Figure 3d shows the computed $\kappa_{L}$ as a function of temperature for the nine compounds A_2_CdX (A = Li, Na, and K; X = Pb, Sn, and Ge). Near room temperature, all compounds exhibit a $\kappa_{L}$ below 1.0 W/mK, which is in very good agreement with our ML predictions. As the temperature increases, $\kappa_{L}$ further decreases as more phonons are excited and cause additional phonon scattering, leading to a reduction in $\kappa_{L}$. These results reveal that *the nine compounds under study are all low-$\kappa_{L}$ materials for potential thermal management and insulation applications*. We further note that our reported $\kappa_{L}$∼0.3 W/mK for K_2_CdPb and K_2_CdSn is consistent with previous DFT studies [80]. Meanwhile, we also found that K_2_CdGe exhibits a comparable theoretical $\kappa_{L}$∼0.3 W/mK. This result is not surprising, given the chemical similarity of the Pb, Sn, and Ge elements. Likewise, for the Li- and Na-based compounds explored here, while our DFT calculations may underestimate their $\kappa_{L}$ values, they are also anticipated to be low-lattice-thermal-conductivity materials based on their chemical similarity to the K-based compounds. In fact, depending on carrier concentration and the underlying temperature, materials with ultra-low $\kappa_{L}$∼0.1 W/mK or lower have been reported in the literature [81,82,83,84]. It would be an important future task to verify our predictions both theoretically (e.g., with different DFT functionals and supercell sizes) and experimentally, through the potential synthesis and characterization of the proposed materials.

Before discussing other thermoelectric properties, we address the small discrepancies between the ML and DFT results in Table 1. First, we note that the ML models were trained to predict $\log(\kappa_{L})$ rather than $\kappa_{L}$ itself. Therefore, a small error in the logarithmic value can be amplified in the actual value. Second, the ML prediction in Table 1 is generally slightly larger than the DFT calculation. One reason for this discrepancy is likely due to the training data distribution. Specifically, while we were interested in discovering materials with low thermal conductivity (i.e., $\log(\kappa_{L})\leq 0$), most of the training data in Figure 1a exhibit $\log(\kappa_{L})\geq 0$. Therefore, one could potentially enhance the ML prediction accuracy by training a weighted ML model. Indeed, when we trained additional ML models by weighting the training samples with $\log(\kappa_{L})\leq 0$ by a factor of 5 to 10, the predicted $\kappa_{L}$ values became smaller and aligned more closely with the DFT results, as seen in Table 1. Notably, the performance of an ML model with a weight factor of 10 is not better than that with a weight factor of 5. Therefore, the sample weight factor has an optimal range and cannot be increased indefinitely. Finally, we note that some ML predictions from the weighted ML models remained larger than the DFT results (e.g., for the sodium compounds in Table 1). This discrepancy may be attributed to the fact that tree models only interpolate so cannot predict values beyond the range of the training dataset. The apparent, albeit small, differences between the ML and DFT predictions are likely associated with the above factors.

**Figure 4:**
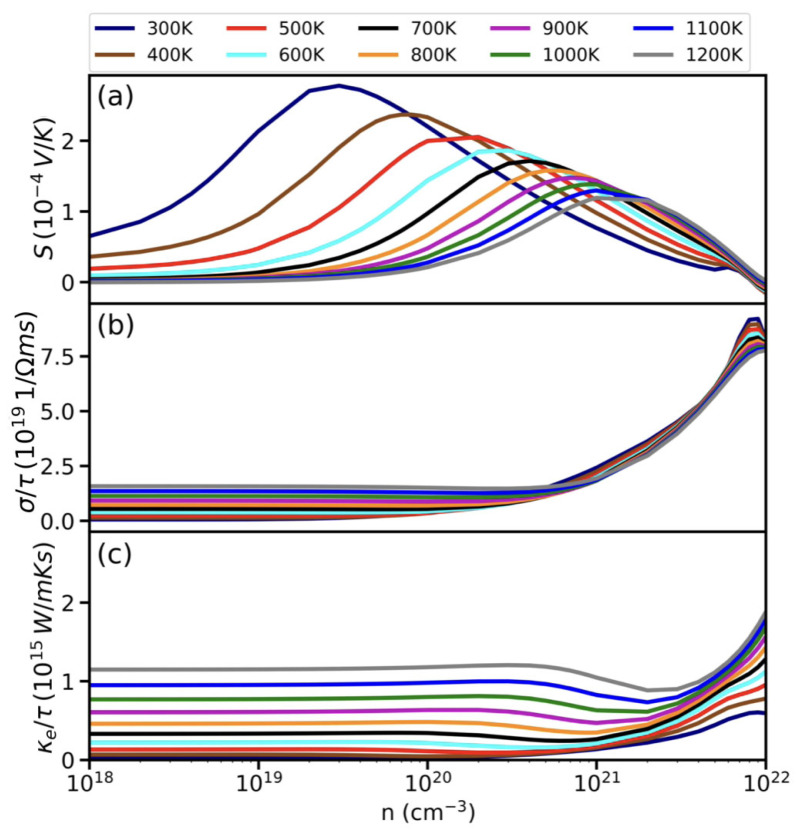
Thermoelectric properties of K_2_CdPb from first-principle calculations: (**a**) Seebeck coefficient (*S*), (**b**) electrical conductivity divided by the relaxation time (σ/τ), (**c**) electronic thermal conductivity divided by the relaxation time ($\kappa_{e}$/τ), as a function of the carrier concentration *n* (in log scale) over the temperature range of 300–1200 K.

We next turn our attention to the thermoelectric properties. Figure 4 displays the DFT calculations for K_2_CdPb: Seebeck coefficient *S* [panel (a)], electrical conductivity divided by the relaxation time σ/τ [panel (b)], and electronic thermal conductivity divided by the relaxation time $\kappa_{e}/\tau$ [panel (c)], as a function of the carrier concentration *n* in the temperature range 300–1200 K. In general, the Seebeck coefficient exhibits a more complex temperature and carrier concentration dependence, but its behavior can be understood qualitatively by considering that of a simple parabolic band [85,86]:

(5) $$S=\frac{8\pi^{2}k_{B}^{2}}{3eh^{2}}m^{*}T{\left(\frac{\pi }{3n}\right)}^{2/3}.$$

Here, $k_{B}$, *e*, *h*, and $m^{*}$ are the Boltzmann constant, electron charge, Plank constant, and carrier effective mass, respectively. Equation (5) dictates that a higher temperature *T* or a lower carrier concentration *n* would result in a larger Seebeck coefficient *S*. These *T* and *n* dependences are indeed consistent with those shown in Figure 4a, especially in the high-carrier-concentration regime ($n>10^{20}$ cm^−3^). In contrast, the low-concentration regime exhibits an opposite trend, where *S* is reduced at higher temperatures. This anomalous behavior is caused by the bipolar effect [87,88,89], where thermal excitations generate both electrons and holes, which contribute opposite signs and lead to an overall reduced *S*.

The behavior of the electrical conductivity σ shown in Figure 4b is more straightforward. Specifically, σ is anticipated to correlate with $n/m^{*}$ and show only weak temperature dependence. Additionally, the electronic thermal conductivity $\kappa_{e}$ can be related to σ via the Wiedemann–Franz law [19]: $\kappa_{e}=L\sigma T$, where *L* is the Lorentz number ($2.44\times 10^{-8}$ WΩ/K^2^ for free electrons). Figure 4c shows that $\kappa_{e}$ roughly exhibits a linear relationship with respect to *T* and *n*, which indeed closely follows the Wiedemann–Franz law. We note that $\kappa_{e}$ becomes significantly larger only near *n*∼$10^{22}$ cm^−3^ or at high temperature. It remains computationally very challenging to directly compute the relaxation time τ from first principles. Meanwhile, in assuming a typical value of $\tau =1\times 10^{-14}$ s (also commonly employed in the literature), $\kappa_{e}$ is less than 1–10 W/mK in most of the temperature range and carrier concentrations under study. Thus, K_2_CdPb remains a low-κ material even after taking into account the electronic contribution.

**Figure 5:**
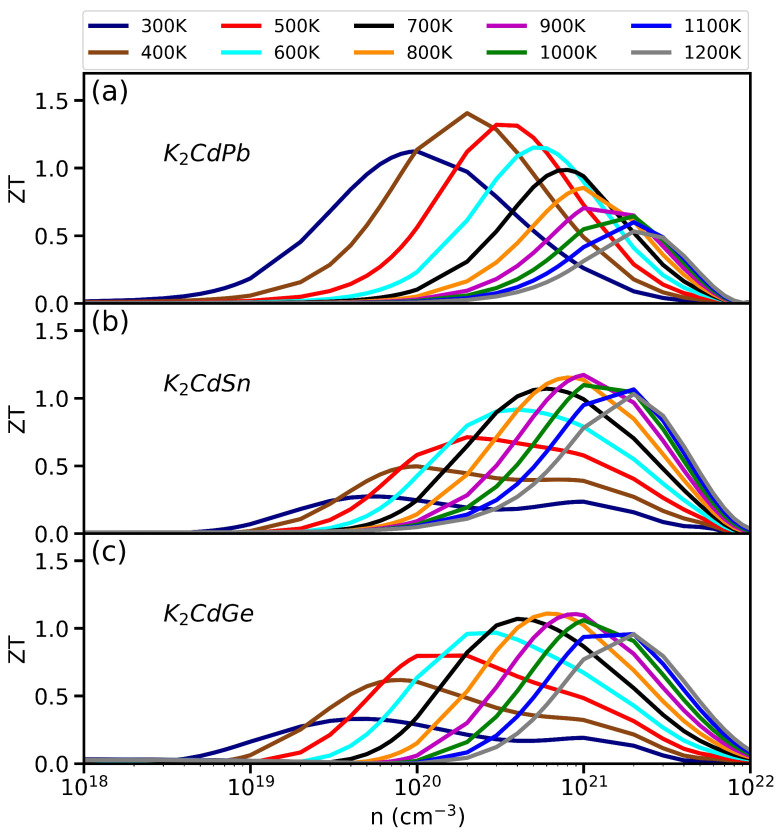
ZT values of thermoelectric performance from first-principle calculations for (**a**) K_2_CdPb, (**b**) K_2_CdSn, and (**c**) K_2_CdGe as a function of the carrier concentration *n* (in log scale) over the temperature range of 300–1200 K.

Finally, since low-κ materials can be good candidates for thermoelectric applications, we also computed their figure of merit, $ZT=S^{2}\sigma T/\kappa$, where the thermal conductivity $\kappa =\kappa_{e}+\kappa_{L}$ includes both electronic and lattice contributions. Figure 5a–c show the ZT values, respectively, for K_2_CdPb, K_2_CdSn, and K_2_CdGe, as functions of carrier concentration *n* (in log scale) and temperature *T*. In all three compounds, the ZT values can exceed 1.0. As an example to estimate the ZT value, for K_2_CdPb at T=400 K and $n=2\times 10^{20}$ cm^−3^, the relevant parameters from our calculations are *S*∼$1.8\times 10^{-4}$ V/K, σ∼$0.1\times 10^{5}$ 1/Ωm, and $\kappa =\kappa_{e}+\kappa_{L}$∼1.0 W/mK. Together, these values lead to a figure of merit $ZT=S^{2}\sigma T/\kappa (∼$$3.24\times 10^{-8}\times 0.1\times 10^{5}\times 400/1.0)$∼1.3–1.4 for K_2_CdPb, making it a *promising low-temperature thermoelectric material*. In contrast, K_2_CdSn and K_2_CdGe show peak ZT values of ∼1.1 near $n=9\times 10^{20}$ cm^−3^ and T=900 K, and they are more suitable for thermoelectric applications at higher temperatures. For the other compounds based on sodium and lithium listed in Table 1, the ZT values are less than 1.0, making them unsuitable for practical thermoelectric applications. However, they could still be potential candidates for thermal insulation materials.

## 4. Conclusions

We developed machine learning (ML) models using Random Forest to efficiently predict the lattice thermal conductivity ($\kappa_{L}$) of a given chemical compound. We also conducted first-principle density functional theory (DFT) calculations to validate the ML predictions. The results indicate that the nine Zintl-phase Cd compounds A_2_CdX (A = Li, Na, and K; X = Pb, Sn, and Ge) with orthorhombic crystal symmetry all exhibit very low lattice thermal conductivity, with $\kappa_{L}\leq 1.0$ W/mK. Our DFT calculations of the figure of merit, ZT, for thermoelectric performance further showed that K_2_CdPb exhibits a peak ZT∼1.4 near 400 Kelvin, making it a promising low-temperature thermoelectric material. Additionally, K_2_CdSn and K_2_CdGe were found to display ZT values of ∼1.1 at 900 Kelvin, suggesting that they could be candidate thermoelectric materials at higher temperatures.

For Li_2_CdX and Na_2_CdX (X = Pb, Sn, and Ge), the ZT values are less than 1.0, indicating more limited practical thermoelectric applications. Nevertheless, their ultra-low lattice thermal conductivity make these materials potentially useful for thermal management and insulation applications. Overall, our study demonstrated that data-driven ML methods are powerful tools for large-scale materials modeling and discovery. The experimental verification of our ML and DFT predictions on the thermoelectric properties of the Zintl-phase Cd compounds would be an important next step. Further theoretical exploration of additional low-$\kappa_{L}$ and high-ZT materials using a combined ML and DFT methodology will continue to be an important area of future research.

## Figures and Tables

**Table 1:** Machine learning (ML) and density functional theory (DFT) predictions of the lattice thermal conductivity $\kappa_{L}$ (in units of W/mK) for different Cd compounds. The ML models are based on Random Forest. The terms “ML + Weight 5” and “ML + Weight 10” indicate a weighting factor of 5 and 10, respectively, on samples with $\log(\kappa_{L})\leq 0$ when training the ML models, which places more weight on the low-$\kappa_{L}$ materials.

Methods	K_2_CdPb	K_2_CdSn	K_2_CdGe	Na_2_CdPb	Na_2_CdSn	Na_2_CdGe	Li_2_CdPb	Li_2_CdSn	Li_2_CdGe
ML	0.69	0.79	0.8	0.84	0.76	0.87	0.95	0.71	0.77
DFT	0.295	0.346	0.323	0.022	0.119	0.086	0.032	0.215	0.028
ML + Weight 5	0.56	0.35	0.37	0.45	0.63	0.119	0.68	0.27	0.4
ML + Weight 10	0.469	0.29	0.62	0.43	0.37	0.58	0.76	0.4	0.22

## Data Availability

The data for training machine learning (ML) models and the resulting ML code for predicting lattice thermal conductivity can be found online at the following weblink: https://github.com/CMLUAB/ML_lattice-themal-conductivity (accessed on 29 October 2024). The data for first-principle calculations are available upon request from the authors.
